# Molar Tooth Sign with Deranged Liver Function Tests: An Indian Case with COACH Syndrome

**DOI:** 10.1155/2015/385910

**Published:** 2015-05-17

**Authors:** Rama Krishna Sanjeev, Seema Kapoor, Manisha Goyal, Rajiv Kapur, Joseph Gerard Gleeson

**Affiliations:** ^1^Department of Pediatrics, ACMS, India; ^2^Division of Genetics, Lok Nayak & Maulana Azad Medical College, New Delhi, India; ^3^Department of Paediatrics, Maulana Azad Medical College, New Delhi, India; ^4^Department of Radiology, ACMS, New Delhi, India; ^5^Neurogenetics Laboratory, Department of Neurosciences and Paediatrics, USA; ^6^Rady Children's Hospital, USA; ^7^Howard Hughes Medical Institute, CA, USA

## Abstract

We report the first genetically proven case of COACH syndrome from the Indian subcontinent in a 6-year-old girl who presented with typical features of Joubert syndrome along with hepatic involvement. Mutation analysis revealed compound heterozygous missense mutation in the known gene *TMEM67* (also called MKS3).

## 1. Introduction

COACH syndrome (cerebellar vermis hypo/aplasia, oligophrenia, congenital ataxia, coloboma, and hepatic fibrosis; OMIM# 216360) is a rare autosomal recessive multisystemic disorder first proposed by Verloes and Lambotte [1]. Joubert syndrome (JS) is characterized by the molar tooth sign (MTS), hypotonia, developmental delay, ataxia, irregular breathing pattern, and abnormal eye movements. COACH syndrome is considered by some to be a subtype of Joubert syndrome [2–4]. Mutations in the* TMEM67* gene are responsible for the majority of COACH syndrome, with minor contributions from CC2D2A and RPGRIP1L [5]. Our patient had compound heterozygous mutation in the* TMEM67* gene.

## 2. Case Summary

A 6-year-old girl presented with global developmental delay, large head, and ataxic gait. The proband was the only child born to nonconsanguineous couple at term after LSCS with birth weight of 3.5 kg. Her motor developmental milestones and speech were grossly delayed.

On examination, her weight was 18.2 kg (50th centile), height 107 cm (50th centile), and head circumference 53 cm (75th–95th centile). Facial features revealed large head, frontal bossing, and hypertelorism with squint. There was pectus excavatum, bilateral clinodactyly, and hepatomegaly (liver span: 10 cm). Neurologically, there was ataxic gait with generalized hypotonia. Eye evaluation showed squint in the right eye (30-degree exotropia) with normal fundus.

**Figure 1:**
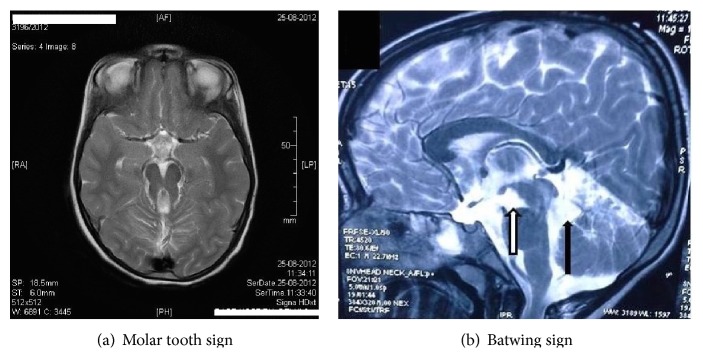
(a) shows thinning of the isthmus with hypoplastic superior cerebellar peduncles forming typical “molar tooth appearance.” (b) Hypoplasia of vermis with resultant “batwing appearance” in the fourth ventricle.

Investigations showed normal metabolic parameters except elevated liver enzymes (SGPT = 261 U/L; SGOT = 110 U/L) with normal bilirubin (0.68 mg). MRI brain revealed “molar tooth appearance” (Figure 1(a)) and “Batwing appearance” (Figure 1(b)).

In view of MRI findings with deranged liver function test, COACH syndrome was suspected. Mutation analysis showed the patient to have a compound heterozygous missense mutation at position c.1413-1G>A and c.434T>G in the gene* TMEM67* with father and mother being carriers for c.1413-1G>A and c.434T>G, respectively.

## 3. Discussion

Joubert syndrome (JS) is a rare genetic disorder affecting the cerebellum characterised by the absence or underdevelopment of cerebellum and malformed brain stem. Diagnosis is based on neurologic signs of hypotonia, ataxia, developmental delay, and oculomotor apraxia along with the “molar tooth sign” (MTS) on MRI, which is the neuroradiologic hallmark of the condition. Disorders that share the MTS have been termed Joubert syndrome (JS). JS comes under the ciliopathies, which are a group of disorders with genetic mutations encoding defective proteins which result in abnormal formation or function of cilia [6].

Cilia are classified into motile and immotile (primary). All the causative JS genes identified to date encode proteins that are localized to the base or axoneme of the cilium [6]. Combinations of additional features such as polydactyly, ocular coloboma, retinal dystrophy, renal disease, hepatic fibrosis, encephalocele, and other brain malformations are classified as Joubert Syndrome Related Disorder (JSRD) [3]. To date, 22 genes, which account for only half of Joubert syndrome cases, have been identified, all encoding for proteins of the primary cilium [6].

Motile cilia when defective cause primary ciliary dyskinesias, which comprise a heterogenous group of disorders characterised by bronchiectasis, left right asymmetry, and infertility [7]. Immotile or primary cilia are present in many differentiated cells of mammalian body like the kidney cells or neurons. All immotile (primary) cilia can now be viewed as sensory cellular antennae with chemosensory, osmosensory, and phototransduction functions [7]. While any organ can be affected by ciliopathic dysfunction, the eye, brain, kidney, and liver are primarily affected [7].

COACH syndrome (OMIM 21630) is considered by some to be a subtype of Joubert syndrome with congenital hepatic fibrosis. The key feature of COACH is congenital hepatic fibrosis (CHF), resulting from malformation of the embryonic ductal plate [1]. In our patient, we found compound heterozygous mutation in the known gene* TMEM67*. Brancati et al. [8] identified compound heterozygous mutation in* TMEM67* gene.

In our patient, we found hepatomegaly and raised liver enzymes with typical features of Joubert syndrome in MRI (molar tooth sign and batwing sign) suggesting COACH syndrome. Clinically, the liver disease can vary from raised liver enzymes to features of portal hypertension [5]. Identification of liver disease is critical because some may develop portal hypertension with variceal bleeding. Known ocular features in COACH syndrome are nystagmus, strabismus, amblyopia, ptosis, and pigmentary retinopathy, of which strabismus was present.

Renal manifestations, reported in subjects with JSRD, were not present in our case. Thus, the presence of mutation in* TMEM67* gene in this patient further delineates the genotype-phenotype correlation in COACH syndrome. JSRD patients with known liver involvement should be tested first for* TMEM67* mutations, followed by CC2D2A and RPGRIP1L. Presymptomatic, gene based diagnosis should make it possible to deliver improved care for systemic complications and prenatal identification.
